# Design, Production and Quality Assessment of Antioxidant-Enriched Olive Paste Dips Using Agro-Food By-Products

**DOI:** 10.3390/molecules30173459

**Published:** 2025-08-22

**Authors:** Efimia Dermesonlouoglou, Athanasios Limnaios, Ioanna Bouskou, Athina Ntzimani, Maria Tsevdou, Petros Taoukis

**Affiliations:** Laboratory of Food Chemistry and Technology, School of Chemical Engineering, National Technical University of Athens, 5 Heroon Polytechniou Str., 157 72 Athens, Greece; efider@chemeng.ntua.gr (E.D.); alimnaios@chemeng.ntua.gr (A.L.); joannabouskou@gmail.com (I.B.); ntzimani@chemeng.ntua.gr (A.N.); mtsevdou@chemeng.ntua.gr (M.T.)

**Keywords:** olive pomace, tomato pomace, valorization, spread, bioactives, enrichment, quality, shelf life stability

## Abstract

This study focuses on the design, development and quality assessment of an innovative shelf-stable olive paste dip, aiming at the valorization of by-products of tomato processing and olive oil production (Product 1: OPD). Bioactive compounds (BACs), i.e., total carotenoids and phenolic components, were extracted from tomato and olive pomace, respectively. For further enrichment, BACs were incorporated in olive paste dips into a second product (OPDEnr) in encapsulated form (Product 2: OPD_Enr_). The total carotenoids (TC) of OPD and OPD_Enr_ were 20.0 ± 2.0 and 30.2 ± 1.0 mg/kg, respectively. Similarly, the total phenolic content (TPC) and the antioxidant activity (AA) were 1.62 ± 0.08 and 3.05 ± 0.10 mg GAE/g, and 0.801 ± 0.075 and 0.976 ± 0.032 mg Trolox/g, respectively. The quality of the developed olive paste dip product prototypes was assessed using the Accelerated Shelf Life Testing (ASLT) methodology at a temperature range of 20–40 °C. Both OPD_Enr_ and OPD were microbiologically stable during storage (i.e., not exceeding 4 logCFU/g for total mesophilic counts), and no lipid oxidation evolution was observed (Peroxide Value, PV did not exceed 4 meq O_2_/kg), while TC, TPC and AA values remained stable. The shelf life of OPD_Enr_ and OPD was determined based on the overall sensory quality and was found to be 120 and 211 d at 25 °C, respectively. OPD_Enr_ and OPD were characterized by a high quality (color and texture), with an overall sensory score of 8.0/9.0 and 9.0/9.0, respectively, in the acceptability–hedonic scale 1 (dislike extremely)-9 (like extremely), and they could potentially be consumed as an antioxidant-enriched olive paste dip.

## 1. Introduction

The food industry is a major contributor to global waste generation across the supply chain, making by-product valorization a critical sustainability priority. It is estimated that approximately 1.3 billion tons of food are wasted annually worldwide, with a significant portion originating from the processing of fruits and vegetables (FAO, 2011). Among these, the tomato and olive oil sectors generate large volumes of by-products with high potential for reuse.

Globally, approximately 180 million tons of tomatoes are processed annually—400,000 tons in Greece alone—producing side streams such as skins, seeds, pulp, and spoiled fruits (World Processing Tomato Council, 2020). Tomato pomace (TP), mainly composed of skins and seeds, accounts for 5–10% of the total tomato mass [1,2]. Although commonly used as fertilizer or animal feed, TP contains valuable bioactive compounds, including lycopene, β-carotene, and phenolic compounds [3], which could be extracted for use in functional food products.

Similarly, the olive oil industry—dominated by Mediterranean countries—generates substantial waste, with the EU alone producing around 8.4 million tons of olive pomace (OP) annually [4]. Greece ranks among the top three producers, contributing 13% of EU production and the highest per capita consumption (~12 kg/year) [5,6]. OP, composed of skin, pulp, stones, and kernels, retains approximately 45% of the phenolic compounds originally present in the olives compared to only 1–2% that enter the oil phase [7,8]. Despite this, OP is still primarily used in low-value applications such as compost or animal feed, though it has demonstrated potential for high-value food and nutraceutical applications due to its antioxidant, anti-inflammatory, and cardioprotective properties [9,10,11].

Current valorization strategies for TP and OP—such as composting, animal feed, biofuels, and soil conditioners—are limited by factors like high moisture content, perishability, and seasonal availability, often resulting in low-value outputs [12,13]. A more sustainable approach involves extracting bioactive compounds for incorporation into functional foods, thereby enhancing nutritional quality while reducing waste [14,15,16,17].

However, the effectiveness of these bioactives in food applications is often hindered by poor solubility, low bioaccessibility, and instability during processing and storage [18,19]. To address the challenges associated with delivering bioactive compounds in food systems, a range of technological solutions have been explored, including chemical modification [20] and biopolymer-based encapsulation techniques [21]. In recent years, the integration of emerging technologies with conventional encapsulation methods has led to the development of advanced delivery systems—such as nanoparticles, nanoemulsions, liposomes, hydrogels, and solid lipid nanoparticles—with diverse properties and functionalities [22,23]. These systems enable the encapsulation of one or more bioactive compounds, enhancing their solubility, bioavailability, and stability using lipid, surfactant, and biopolymer-based matrices [24]. Furthermore, such carriers offer controlled or sustained release and protect sensitive compounds from environmental stressors during food processing and storage. The design and selection of optimal formulations are crucial for the successful application of nanocarrier systems in food product development. Advanced delivery systems, such as nanoemulsions, offer a promising solution. By encapsulating these compounds in nano-sized droplets, nanoemulsions improve solubility, stability, and bioavailability while preserving both sensory and nutritional qualities throughout the shelf life of food products [25,26,27,28,29,30]. Microencapsulation technology enhances the stability of encapsulated components by limiting their interaction with the external environment. This approach can improve resistance to environmental factors, mask undesirable tastes and odors, facilitate the transport and storage of volatile substances such as liquids and gases, and enable the controlled release of the active components [30].

Despite growing research on bioactive extraction and delivery systems, few studies have focused on incorporating stabilized pomace-derived compounds into traditional food products. This gap limits the practical application of these sustainability strategies in consumer-facing innovations.

Olive pastes (green and/or black) are traditional Mediterranean spreads made from finely crushed olives preserved in extra virgin olive oil and may be enriched with ingredients such as herbs, sun-dried tomatoes, capers, or artichokes [31]. Their quality is influenced by raw materials and processing methods. For example, pasteurization—used to extend shelf life—may alter chemical composition, leading to color changes (e.g., via Maillard reactions) and flavor modifications, including the development of cooked notes that affect sensory characteristics [32,33,34]. Fortifying such products with stabilized bioactives from pomace could enhance their nutritional value and functional appeal.

The aim of this study was to design and develop innovative olive paste dip products of extended shelf life based on by-products of tomato processing and olive oil production, specifically tomato skins and seeds and olive pomace, to valorize these significant by-products of the food industry and align with circular economy principles. The olive paste dip prototypes were also enriched with bioactive compounds (BACs) extracted from the aforementioned food industry by-products, specifically phenolics and carotenoids, to further enhance their antioxidant and bio-functional properties, increasing their nutritional value. The quality and stability (i.e., sensorial, physicochemical, and microbiological stability) of the developed olive paste dip prototypes was monitored during storage in a wide range of temperatures (from 20 to 40 °C), and their shelf life was calculated.

## 2. Results and Discussion

### 2.1. Product Characterization

The results for the physicochemical, nutritional and quality properties of the developed olive paste dip product prototypes OPD and OPD_Enr_ are presented in Table 1. A comparison of the properties of the two products OPD and OPD_Enr_ revealed no significant differences in moisture content, ash content, pH, and water activity (*p* > 0.05). The product enriched with BACs (OPD_Enr_) exhibited higher titratable acidity (TA%) values (*p* < 0.05) due to the addition of the encapsulated BACs. Textural analysis indicated that the non-enriched product (OPD) was firmer (*p* < 0.05). As expected, the OPD_Enr_ product showed increased initial levels of total carotenoids (TC) and phenolic compounds (TPC), as well as higher antioxidant activity (AA) (*p* < 0.05). These properties led to darker, more yellow-red product color (increased *L*, *a* and *b* values) of the OPD_Enr_ product compared to the OPD product. Similar initial values for the measured physiochemical and quality properties (such as pH, color) were reported Andreou et al. [16], Schwartz et al. [35] and Göktepe et al. [31]. For example, Andreou et al. incorporated olive paste residue in an innovative, high-added-value, healthy olive-based spread, along with other functional ingredients such as honey and ground nuts with similar properties such as pH (4.23), a_w_ (0.941), moisture content (49.15%), ash content (0.93%), and crude fibers (>6 g/100 g of the product) [16]. Regarding the proximate analysis results (i.e., fibers, proteins, carbohydrates, salt, energy), the two products, OPD_Enr_ and OPD, presented the same profile due to the same composition.

### 2.2. Shelf Life Determination

#### 2.2.1. Evolution of Color and Texture of Olive Paste Dip Products During Storage

**Figure 1:**
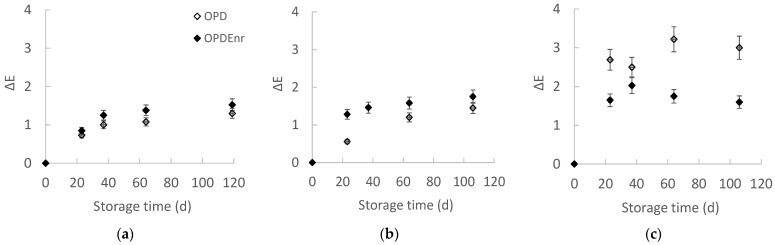
Evolution of color with storage time at (**a**) 20, (**b**) 30 and (**c**) 40 °C for products OPD and OPD_Enr_ (mean value of three ± standard deviation).

Figure 1a–c presents the evolution of the total color difference Δ*E* for the developed olive paste dip products OPD and OPD_Enr_ with the storage time at all storage temperatures. Total color difference (Δ*E*) value, which was calculated based on the color of the initial product (storage day 0), increased during the storage of both samples. Δ*E* values were low, ranging from 0 to 3 (the highest value was calculated for the OPD_Enr_ stored at 40 °C for 60 d). The untrained eye may only be able to see a visual difference between two samples of color that have a Δ*E* of 5 or more, while in the range 1 < Δ*E* < 2, only highly trained observers can see differences [36]. A significant temperature-dependent color change was observed from 20 to 40 °C. Products stored at 30 and 40 °C showed statistically significant differences (*p* < 0.05). At 40 °C, there are significant differences between OPD and OPD_Enr_; OPD_Enr_ exhibited lower Δ*E* values compared to OPD (*p* < 0.05) showing that the addition of BACs led to color stability. Similarly, Stoll et al. found that both free and microencapsulated thyme essential oil helped maintain stable L*, a*, and b* color values in olive paste during storage [37]. Jafari et al. attributed color changes to pigment instability linked to increasing pH but observed that samples treated with free and encapsulated olive phenolic extract showed the lowest color degradation [38]. Olive leaves are rich in phenolic compounds, primarily oleuropein, along with flavonoids and secoiridoids, which exhibit strong antioxidant activity. This antioxidant capacity contributes to pigment protection and color stability during storage [39].

**Figure 2:**
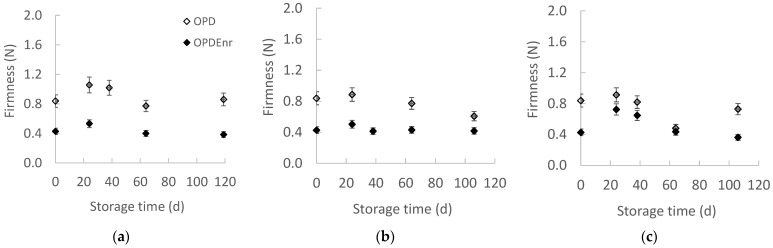
Evolution of texture with storage time at (**a**) 20, (**b**) 30 and (**c**) 40 °C for products OPD and OPD_Enr_ (mean value of three ± standard deviation).

Figure 2a–c presents the evolution of the firmness for the developed olive paste dip products OPD and OPD_Enr_ with the storage time at all storage temperatures. Statistically significant differences were observed between the samples at all three temperatures (*p* < 0.05). It appears that OPD_Enr_ product was less firm compared to the OPD one. No specific trend was observed over time; both OPD_Enr_ and OPD_Enr_ remained almost stable throughout the experiments (*p* > 0.05).

#### 2.2.2. Evolution of Total Carotenoids of Olive Paste Dip Products During Storage

**Figure 3:**
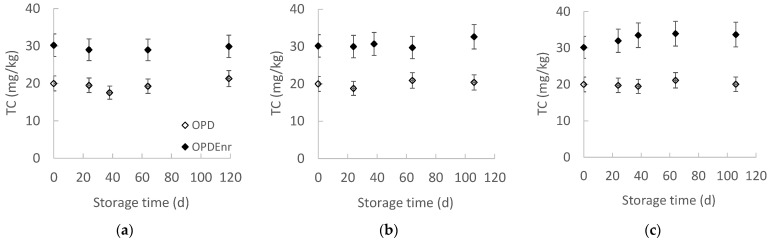
Evolution of total carotenoids with storage time at (**a**) 20, (**b**) 30 and (**c**) 40 °C for products OPD and OPD_Enr_ (mean value of three ± standard deviation).

Figure 3 presents the evolution of total carotenoid concentration for the developed olive paste dip product prototypes OPD and OPD_Enrc_ with the storage time at all storage temperatures. The initial carotenoid concentration remained relatively stable throughout the storage period. Specifically, the non-enriched product OPD exhibited an initial concentration (C_0_) of 20.0 mg TC/kg product, while the enriched product OPD_Enr_ showed a higher initial concentration of 30.2 mg TC/kg product. The statistical analysis results showed that there were no statistically significant changes in carotenoid content over storage time (*p* > 0.05). The carotenoid content in the tomato extract was 1.38 g per 100 g of dry capsules. Furthermore, the carotenoid concentration in OPD_Enr_ was consistently higher than that in OPD, attributable to the addition of carotenoid-rich extracts. This difference was statistically significant across all storage temperatures evaluated (*p* < 0.05).

#### 2.2.3. Evolution of Total Phenolic Content (TPC) and Antioxidant Activity (AA) of Olive Paste Dip Products During Storage

**Figure 4:**
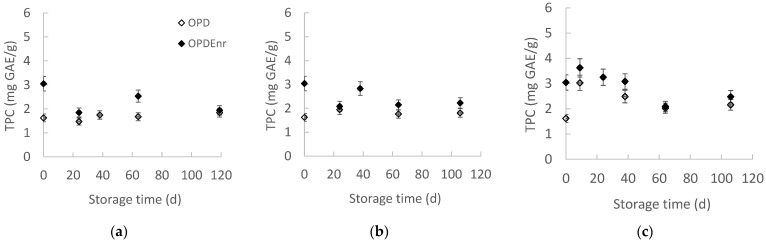

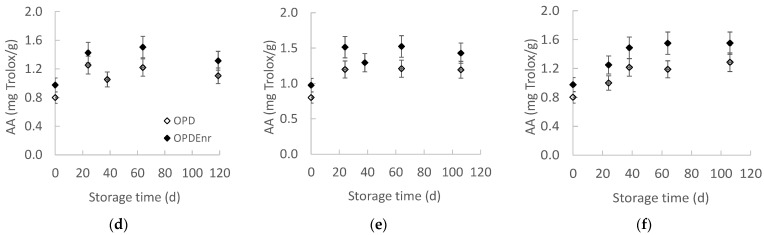
Total phenolic content (TPC) (**a**–**c**) and antioxidant activity (AA) (**d**–**f**) of products, OPD and OPD_Enr_, with storage time at (**a**,**d**) 20, (**b**,**e**) 30 and (**c**,**f**) 40 °C (mean value of three ± standard deviation).

The evolution of TPC as a function of storage time was examined at all storage temperatures, presented in Figure 4a–c. Τhe phenolic compounds had an initial tendency to decrease, and then they remained stable. TPC of OPD and OPD_Enr_ products did not show statistically significant differences (*p* > 0.05), apart from the TPC values of products stored at the highest temperature of 40 °C. The initial TPC of OPD was C_0_ = 1.62 mg GAE/g compared to the C_0_ = 3.05 mg GAE/g of OPD_Enr_, which means that the addition of BACs led to an increase in the initial concentration of phenolic compounds. At all temperatures, the OPD_Enr_ samples presented higher TPC values, showing the positive effect of encapsulation on the stability of the concentration of phenolic compounds. Karagozlu et al. reported total phenolic content (TPC) values ranging from 3.568 to 5.971 mg/g in olive paste samples stored at 4–25 °C, with the highest TPC observed in enriched (via encapsulation) TEO samples at 4 °C at the start of storage. TPC declined over time, particularly at higher temperatures, likely due to phenolic degradation [40]. Mahungu et al. attributed this to the temperature-induced decarboxylation of malonyl groups [41].

The evolution of antioxidant activity (AA), using the DPPH method, as a function of the storage time was examined at all storage temperatures, presented in Figure 4d–f. Statistically significant differences (*p* < 0.05) were observed between the samples at all three storage temperatures, with the enriched samples, OPD_Enr_, exhibiting higher antioxidant values than the non-enriched ones, OPD. At storage time 0, the antioxidant activity measured 0.80 mg Trolox/g for OPD and 0.98 mg Trolox/g for OPD_Enr_. This difference is attributed to the presence of extra BACs, which enhanced the antioxidant activity of the OPD_Enr_ samples. The OPD sample contains fewer phenolic compounds compared to the samples containing encapsulated BACs (OPD_Enr_). Over time, a gradual increase was recorded. At the end of the storage, 119 days, at 20 °C, antioxidant levels increased by 38% in the OPD samples and by 35% in the OPD_Enr_ ones. After 106 days at 30 °C, increases of 49% and 46% were calculated for the OPD and OPD_Enr_ samples, respectively. At 40 °C, the most pronounced increase was observed, with values increasing by 61% in the OPD samples and 59% in the OPD_Enr_ samples after 106-day storage. This observation can be attributed to several factors. The most probable reason is the gradual release of the antioxidant phenolics and carotenoids from the matrix of the encapsulation system, resulting in an increase in the antioxidant activity of the product. However, additional factors should be considered for the gradual increase in the antioxidant activity of the product since the high moisture of the product could quickly destroy the encapsulation matrix and significantly accelerate the release of the BACs. Another possible explanation for the increase in the antioxidant activity of the product could be attributed to the activity of the enzymes and microorganisms (e.g., lactic acid bacteria) involved in the fermentation or ripening of the olive paste dips, which could release or activate more polyphenols that result in an increase in the antioxidant activity of the product [42]. Such processes are known to accelerate at higher temperatures, around 40 °C, which could explain the higher antioxidant activity values at higher storage temperature. Additionally, the Maillard reaction of peptides and sugars present in the olive paste dips forming products with high antioxidant activity should be considered. The Maillard reaction is also enhanced by the increase in temperature, which could explain the higher antioxidant activity values at higher storage temperatures, as well [43,44]. Göktepe et al. and Karagözlu et al. reported similar AA values for olive paste samples during their storage [30,40]. Cam et al. reported that the addition of pomegranate peel phenolics to ice cream achieved a significant increase in the AA of ice creams compared to non-enriched, control samples [45].

#### 2.2.4. Evolution of Lipid Oxidation of Olive Paste Dip Products During Storage

**Figure 5:**
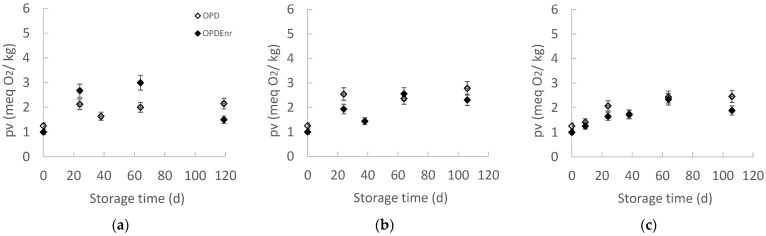
Peroxide Value (pv, meq O_2_/kg) of oil extracted from products, OPD and OPD_Enr_, with storage time at (**a**) 20, (**b**) 30 and (**c**) 40 °C (mean value of three ± standard deviation).

Peroxides are the primary oxidation products of fats, which are not perceptible sensorially since they are tasteless and odorless but show high toxicity and promote biological oxidations in the human body. Therefore, the concentration of peroxides is one of the most important indicators of the quality of fats and oils [46]. The maximum acceptable Peroxide Value (PV) for fats and oils, was set at 20 meq O_2_/kg oil [47]. PV limits appear to vary by product category; therefore, they can be determined by correlating consumer acceptance with PV number trends during storage. In the case of olive oil and products, PV = 5.5–6.5 is considered low [48]. The evolution of the PV of OPD and OPD_Enr_ products at three temperatures (20, 30, and 40 °C) is presented in Figure 5. The highest PV recorded was 4 meq O_2_/kg, indicating that no significant oxidation has occurred in the products. Statistically significant differences over time were observed at all three temperatures (*p* < 0.05). At 20, 30, and 40 °C, the enriched with BAC samples, OPD_Enr_, exhibited lower PVs compared to non-enriched OPD samples. This is likely due to the antioxidant effect provided by the added bioactive compounds. Phenolic compounds, in particular, help protect the oils against oxidation. Göktepe et al. reported an increase in the PV of olive paste from 2.12–2.25 to 21.01–39.16 meq O_2_/kg over 56 d, with essential oils more effectively delaying oxidation [30]. Similarly, Schwartz et al. observed a PV rise from 5 to 27 meq O_2_/kg without the sensory detection of rancidity [35]. Karaaslan et al. found stable PVs (6.35 to 6.37 meq O_2_/kg) in enriched pepper seed oil over four weeks, emphasizing that enrichment with pepper seed oil contributed to oxidative stability [49].

#### 2.2.5. Evolution of Microbial Load During Storage

No significant growth of microorganisms was observed for products OPD and OPD_Enr_ during their storage at the three temperatures (*p > 0.05*). Both products were microbiologically stable (up to 119 days) as the logCFU values for TMVC were particularly low throughout the shelf life experiment. In Table 2, the evolution of logCFU values for TMVC during storage were presented. Yeasts, molds, lactic acid bacteria and *pseudomonads* were not detected during storage.

#### 2.2.6. Evolution of Sensory Properties of Olive Paste Dip Products During Storage

**Figure 6:**
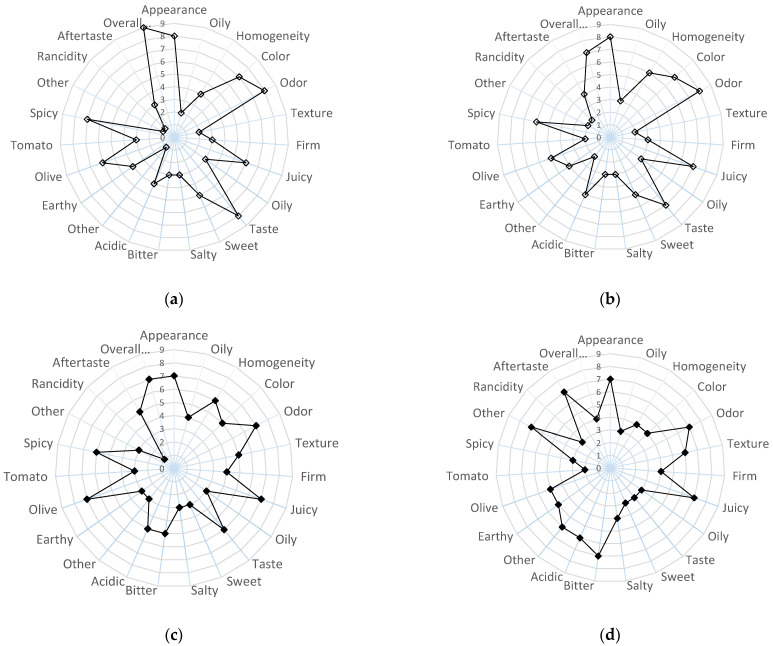
Average scores for sensory properties for the products, OPD (**a**,**b**) and OPD_Enr_ (**c**,**d**), initially (**a**,**c**) and at storage time t = 104 d at 20 °C (**b**,**d**).

The sensorial quality of the products, OPD and OPD_Enr_, can be described in terms of appearance (oil detection and color browning), odor, texture (crispiness, firmness, and gumminess), taste (overall, sweet taste, salty taste, and other), flavor, aftertaste, and total sensory quality. In Figure 6, average scores (scale 1–9) for the characteristics (axes 1–23) of OPD_Enr_ and OPD_Enr_ products are demonstrated.

At zero time (Figure 6), the product enriched with BACs (OPD_Enr_) demonstrated greater visual homogeneity (6/9 vs. 4/9) and more intense oil detection (4/9 vs. 2/9) compared to the non-enriched sample (OPD) (*p* < 0.05). Textural differences were noted both visually and orally, with product OPD_Enr_ perceived as slightly firmer (4/9 vs. 3/9) and juicier (7/9 vs. 6/9) (however, no statistically significant differences *p* > 0.05 were revealed). In terms of taste, enrichment led to increased acidity (5/9 vs. 3/9), bitterness (5/9 vs. 3/9), and reduced sweetness (3/9 vs. 5/9), along with a stronger olive (7/9 vs. 6/9) aroma (*p* < 0.05). However, the intensified aroma profile in the OPD_Enr_ product negatively impacted overall liking (8/9 vs. 9/9) (*p* < 0.05). Over storage time, the OPD_Enr_ product showed a decline in desirable aromas (olive: 5/9; tomato: 2/9; and pepper: 3/9) and an increase in spoilage-related odors (spoiled: 7/9 and oxidized: 3/9), with a notable impact on aftertaste (7/9) (*p* < 0.05). Consequently, its overall sensory score dropped from 8/9 to 5/9, approaching the acceptability threshold. In contrast, the OPD product remained more stable (score: 9/9 to 7/9), retaining sensory acceptability throughout storage. A sensory score of 5 was established as the minimum threshold for acceptability, below which the product was not sensorially accepted. No statistically significant differences were calculated for scores for appearance, color, odor, taste (sweet or salty), aroma (earthy), and texture (firm, juicy, or oily) characteristics (*p* > 0.05).

The sensory (aroma/flavor, taste, texture, etc.) properties of the olive paste are complex and affect consumer acceptability. Bitterness is primarily associated with polyphenols and affected by debittering techniques. Acidity results from naturally occurring acids (e.g., tartaric, malic, citric) or those produced during lactic fermentation (e.g., lactic, acetic acid) and may also arise from added acids or acidity regulators (e.g., citric or ascorbic acid), including vinegar. Aromatic/spicy notes derive from added spices (e.g., oregano, fennel, thyme, marjoram) or olive variety. Rancid flavor indicates oxidative spoilage. Consistency/firmness reflects resistance to deformation, while oiliness refers to the greasy film perceived in the oral cavity during and after chewing [50].

**Figure 7:**
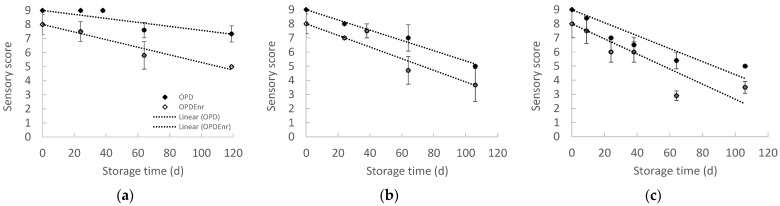
Score for the total sensory quality of products, OPD and OPD_Enr_, with storage time at 20, 30 and 40 °C (mean value of three ± standard deviation).

In Figure 7, the overall sensory acceptance scores of the OPD and OPD_Enr_ samples are representatively presented. It was observed that the enrichment of olive paste dips with bioactive compounds (BACs) extracted from tomato and olive pomace (carotenoids and phenolics, respectively) resulted in lower sensory acceptance scores compared to the non-enriched prototypes. In this study, the formulation of innovative olive paste dip prototypes enriched with the aforementioned BACs was investigated as proof of concept. The quantity of tomato pomace extract added (corresponding to 10 mg lycopene per kg of the final product) was selected in order to reach the maximum permitted concentration of lycopene in the final product, according to European legislation (20–30 mg/kg according to EFSA [51]. For total phenolic compounds (TPC) from olive pomace extract, no legislation restrictions currently exist; hence, a final concentration of 500 mg TPC peg kg of the final product was selected as a TPC concentration offering a significant antioxidant enhancement of the BAC-enriched product (OPD_enr_) based on previous research. The concentration of total phenolic compounds (as gallic acid equivalents) and total carotenoids (as lycopene equivalents) in the olive and tomato pomace extracts was 63.7 mg/g and 7.25 mg/g on a dry basis, respectively. This corresponds to the addition of 7.43 g dried olive pomace extract and 1.38 g dried tomato pomace extract per kg of the final BAC-enriched product (OPD_enr_). Since other compounds with off-flavor characteristics are co-extracted with the phenolics and carotenoids in the olive and tomato pomace extracts and since the quantities of these extracts added to the BAC-enriched olive paste dips ((OPD_Enr_ compared to OPD) are significant, the off-flavors are inevitably transferred to the prototypes. Further investigation and optimization of the olive pomace dip prototypes in the case of commercialization should be carried out to limit the off-flavor characteristics by adjusting formulation or encapsulation strategy.

By fitting the zero-order kinetic model to the experimental data, it was possible to determine the constant rates of sensory deterioration at each storage temperature (*k_s_*). The results of the fitting for the OPD and OPD_Enr_ samples are presented in Table 3.

**Figure 8:**
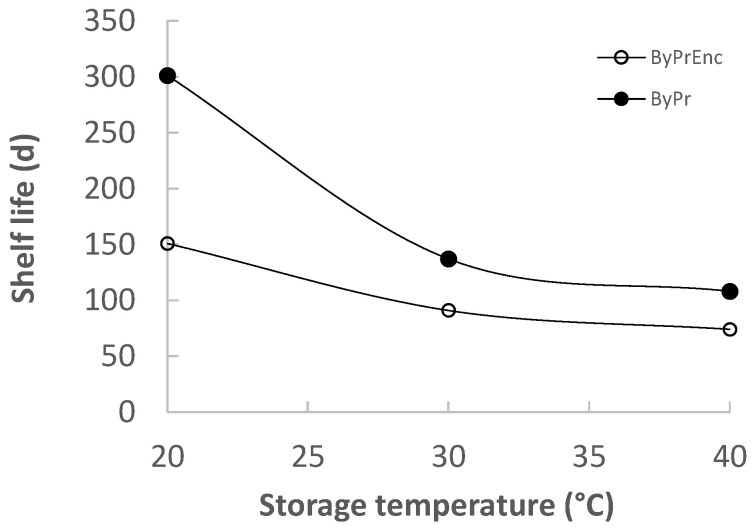
Shelf life—based on sensory deterioration (overall sensory acceptance)—of products OPD and OPD_Enr_ at 20, 30, and 40 °C.

Based on the results obtained from the sensory mathematical modeling, it was possible to determine the shelf life based on the overall sensory quality (Figure 8). Based on the overall sensory quality, the shelf life of OPD_Enr_ and OPD was estimated at 25 °C equal to 120 and 211 d, respectively. The enrichment with additional phenolics and carotenoids (OPD_Enr_) provided higher initial TC, TPC and AA compared to the OPD (initially and throughout the storage), but it negatively affected the product aroma/taste and consequently the overall product acceptability, due to the reasons, resulting in a shorter shelf life.

## 3. Materials and Methods

### 3.1. Materials

The fresh tomato pomace used in this study is a solid residue from the juicing of the industrial tomato hybrid Heinz 3402, sourced from a tomato manufacturing industry in Central Greece. The material consists of approximately 89% *w*/*w* peels and flesh and 11% *w*/*w* seeds, weighed on a dry basis (separated manually). The initial moisture was found to be 88% *w*/*w*. After receiving it, the pomace was air-dried for 24 h at 40 °C (Hotmix Pro air dryer, Modena, Italy), packaged in vacuum-sealed pouches (PET12-ALU8-PE80), and stored at a temperature of -18 °C in the absence of light until use (no longer than 3 months) to prevent the degradation of the material and its contained ingredients.

Fresh olive pomace was sourced from a local olive processing plant carrying out two-phase olive oil extraction from olives of the Manaki variety cultivated in the Peloponnese region in Greece. The raw pomace had a moisture content of 45% *w*/*w* on a wet basis and a residual oil content of 7.4% *w*/*w* on a dry basis. The pomace was kept at 0 °C until further processing. For experiments where dry material was required, the pomace was air-dried (Hotmix Pro air dryer, Modena, Italy) at 40 °C for 24–48 h. The dry pomace was stored in vacuum-sealed multilayered sachets (PET12-ALU8-PE80) at room temperature (for no longer than 1 month).

### 3.2. The Incorporation of BACs into the Product

The target bioactive compounds of the present study include (i) phenolic compounds extracted from olive pomace, with microwave-assisted extraction (60% *v*/*v* MeOH–solid/liquid ratio 1:10 g/mL–300 W–50 °C–5 min), as described by Tsevdou et al. [10] and (ii) carotenoids extracted from tomato pomace, with high-pressure-assisted extraction (ethyl acetate—solid/liquid ratio 1:10 g/mL–650 MPa–25 °C–1 min) followed by conventional extraction with ethyl acetate under stirring conditions (350 rpm–25 °C–24 h). The phenolic compounds and the antioxidant activity in the dry olive extract were measured at 63.7 mg GAE/g and 49.6 mg Trolox/g product, respectively. The concentration of total carotenoids of the extract was determined to be 7.25 mg TC/g.

An oil-in-water nanoemulsion (10% *w*/*w*) enriched with BACs was prepared using a two-stage homogenization process. The content of BACs was 10 mg lycopene/kg of product (EFSA, 2017) and 500 mg phenolics/kg of product [52]. The aqueous phase was prepared by mixing 8% wt Tween 80 using a magnetic stirrer. The lipid phase was prepared by dissolving 1% wt lycopene extract in the pomace oil. Subsequently, the oil phase was homogenized with the aqueous phase at 10,000 rpm for 10 min with a high-speed homogenizer (CAT Unidrive 1000, CATScientific, Paso Robles, CA, USA). Then, the coarse oil in water emulsion was further homogenized with an APV-1000 high-pressure homogenizer (London, UK) in 4 cycles of pressure equal to 600 bar. Finally, the wall material (1:1) was added to the mixture, the mixture was stirred and freeze-dried for 48–72 h to remove the water and we obtained the encapsulated bioactive components in dry form.

### 3.3. Design and Production of Innovative Olive Paste Products

**Figure 9:**
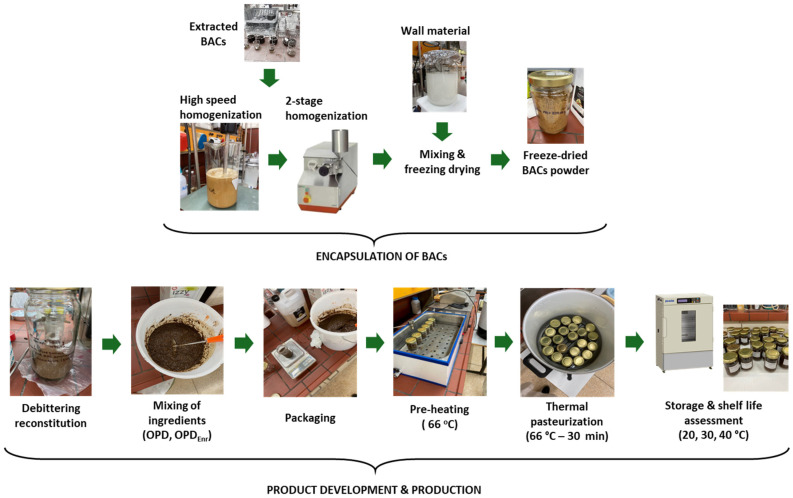
Flow diagram of overall process, from nanoemulsion production to final product development and storage of OPD prototype.

In this study, two innovative olive paste dip prototypes were developed: one enriched with BACs (carotenoids and phenolic components) to enhance product antioxidant properties and increase product stability (OPD_Enr_) and one without enrichment with BACs acting as the control sample (OPD). Dried tomato and olive pomace was incorporated into both olive paste dip prototypes (OPD and OPD_Enr_) to enhance the eco-friendliness of the innovative products via the valorization of food industry by-products. Other ingredients for both products were pomace oil, pepper, olive slices, dry olive by-product, water, vinegar, glycerol, dry tomato pomace, and capers (Table 4). To decrease the bitter flavor (due to oleuropein glucoside, the main phenolic compound of olive oil products/by-products) and increase the sensory quality and acceptability of the product, the debittering of the dry olive pomace was performed (Figure 9) [50,52,53]. The olive paste dip prototypes were packaged in glass jars and thermally pasteurized (66 °C, 30 min).

### 3.4. Determination of Physicochemical Parameters

Olive paste dip products were analyzed for moisture (AOAC 925.40) [54], ash (AOAC 948.22) [55], lipid content (Soxhlet method with ethyl ether; AOAC 945.38) [56], lipids saturated, monosaturated, and unsaturated (AOAC 996.06) [57], total nitrogen content (ISO 1871) [58], total protein (*n* × 6.25), dietary fibers (AOAC 991.43) [59], carbohydrates, sugars (O.613) [60], metal and trace elements, Na (O.521/ICP-MS) [61], and salt (Na × 2.5). The results are expressed as g/100 g as the average of at least three replicates. Water activity was measured using a water activity meter (Lab Touch-Water Activity Meter, Novasina, Lachen, Switzerland)). pH was measured using an AMEL 338 pH meter (Amel Instrument, Milan, Italy).

### 3.5. Other Nutritional and Quality Parameters

#### 3.5.1. Total Phenolic Content (TPC) and Antioxidant Activity (AA) Determination

The Folin–Ciocalteu method, based on a previously reported method with some modifications [62,63], was used to determine the total phenolic content, with gallic acid serving as standard, using a Hitachi U-2900 UV/Vis spectrophotometer (Hitachi, Tokyo, Japan). The results are given as g of gallic acid equivalents (GAE)/kg of dry weight of the product (dw) (g GAE/kg dw). Antioxidant activity (AA) was determined using the DPPH assay. Briefly, after the extraction of total phenolic compounds with 60% methanol solution overnight, 100 μL of appropriately diluted methanol solution extract was mixed with 3.9 mL of a 25 mg/L 2,2-diphenyl-1-picrylhydrazyl (DPPH) solution and incubated for 30 min at ambient temperature in the dark. The absorbance was measured at 515 nm using a U-2900 spectrophotometer (Hitachi, Tokyo, Japan) and the antioxidant activity expressed in terms of Trolox equivalent antioxidant capacity [g Trolox equivalents/kg dry weight of product (dw)] was calculated via the absorbance difference between the sample and the blank using a Trolox standard curve.

#### 3.5.2. Total Carotenoid Determination

The total carotenoid content was measured according to the protocol described by Lichtenthaler & Buschmann [64]. The absorbance of the total carotenoids was measured using a Hitachi U-2900 UV/Vis spectrophotometer (Hitachi, Tokyo, Japan) at 470 nm.

#### 3.5.3. Objective Color and Texture Determination

Objective color was measured on the surface of olive paste on at least five replicates using an Xrite-i1 portable digital colorimeter (Gretag-Macbeth, Grand Rapids, MI, USA) and expressed in the CIE-Lab scale. Color change in samples during storage was expressed using the total color difference, Δ*E*, given by the following equation (Equation (1)):

(1) $$\Delta E=\sqrt{{\left(a-a_{0}\right)}^{2}+{\left(b-b_{0}\right)}^{2}+{\left(L-L_{0}\right)}^{2}}$$

where *L*, *a*, and *b* are the measured CIE-Lab color parameters and *L*_0_, *a*_0_, and *b*_0_ are the measured color parameters of the sample at time zero.

The texture of olive paste was determined using a texture analyzer (TA.XT2i Stable Micro Systems, Godalming, Surrey, UK). Samples were subjected to a double compression test, using a spherical probe of 12.7 mm diameter (P/0.5S, Stable Micro Systems, Godalming, Surrey, UK), with the following set parameters; pre-test speed: 4 mm/s, test speed: 2 mm/s, post-test speed: 2 mm/s, distance: 12 mm, and trigger force: 1 g. All samples were pre-tempered at 25 °C before testing for 3 h. Firmness is the maximum force applied by the probe to fit inside the sample. It is a property describing the slight resistance of the product to deformation. The positive peak of maximum force recorded during the analysis was taken as firmness (*n*) [50]. At least 5 replicates for products were performed per measurement.

#### 3.5.4. Lipid Oxidation: Peroxide Value (PV) Determination

A pre-weighed portion (25.0 g) of olive paste dip product was suspended in 250 mL of hexane, and the mixture was stirred for 2 h to complete the oil extraction. Then, the extract was retrieved via filtration with filter paper. The extract was transferred to a round-bottomed flask, which was connected to a rotary evaporator and placed in a bath at 45 °C, to remove the solvent and obtain the oil. The Peroxide Value (PV) of the resulting oil was measured following the methodology based on the AOCS Cd 8–53 method [65] described by Tsevdou et al. [9], an iodometric method in which hydroperoxides react with hydroiodide and the resulting iodine is titrated with a Na_2_S_2_O_3_ solution.

#### 3.5.5. Determination of Microbial Quality

All microbiological analyses were conducted based on official methods of analysis for total mesophilic viable count (TMVC), yeasts and molds, and lactic acid bacteria [13]. The microbial load of the samples was determined using the surface plating method and the serial 10-fold dilution method, based on growth on agar plates. Samples (0.1 mL) of the appropriate dilutions of the product were spread on the surface and/or poured into the appropriate media in Petri dishes for the enumeration of different spoilage bacteria. For the enumeration of TMVC, the non-selective medium plate count agar (PCA, Biolife, Milan, Italy) was used after incubation at 25 °C for 72 h, whereas the enumeration of yeasts and molds was performed using the selective medium Rose Bengal Chloramphenicol agar (RBC, Neogen, Lansing, MI, USA). The sampling procedure was identical to that of the PCA medium. The plates were incubated for 72 h at 30 °C. Finally, lactic acid bacteria were enumerated on the selective medium deMan, Rogosa, and Sharpe agar (MRS, Merck, Darmstadt, Germany) after incubation at 30 °C for 96 h. After incubation, the colonies were counted, and the microbial load was expressed as the average log CFU/g. *Pseudomonas* spp. were enumerated using spread plate methodology on Cetrimide agar (CFC, Merck, Darmstadt, Germany) after aerobic incubation at 25 °C for 48 h. The detection limit of the microorganisms for the applied methodology was 2.00 logCFU/g and 1.00 logCFU/g for aerobic and facultative anaerobic microorganisms, respectively.

#### 3.5.6. Determination of Sensory Properties

The sensory analysis of the olive paste dip products was carried out using a panel of 8 trained members (each sample tripled, giving a total of 24 assessments per product) in our ISO 17025-accredited sensory laboratory [66,67], which features a standardized testing environment with individual sensory booths [68]. The scored characteristics for products are described in Supplementary Files (Table S1). Samples were presented to the panelist in 3-digit coded containers. To ensure consistency in sensory perception, panelists were instructed to use low-sodium spring water (Zagori, Greece) for palate cleansing. The scored characteristics of the developed products are described as follows: the intensity scale 1–9 (1—lowest intensity–9—highest intensity): appearance (oily or homogenous), color, texture (firm, juicy, or oily), taste (sweet, salty, bitter, acidic, or other), aroma (earthy, olive, tomato, spicy, or other), and rancidity, and the acceptability–hedonic scale 1–9 (1—dislike extremely, 2—dislike very much, 3—dislike moderately, 4—dislike slightly, 5—neither like nor dislike, 6—like slightly, 7—like moderately, 8—like very much, 9—like extremely): appearance, odor, taste, color, after taste, and overall acceptability. Overall sensory quality/liking encompasses the panelists’ general opinion about the samples examined and is usually a good indicator of the evolution of a product’s quality. The dependence of the overall sensory liking on storage temperature is described by the following zero-order equation (Equation (2)):

(2) $$S_{t}=\;S_{0}\;\pm \;k_{s}\cdot t$$

where *S* is the sensory score at time *t*, *S*_0_ is the sensory score at time zero, and *k_s_* is the rate of score deterioration. The dependence of model parameter *k_s_* for the primary model (Equation (2)) on storage was mathematically modeled using the Arrhenius equation (Equation (3)):

(3) $$lnk_{s}=lnk_{s,T_{ref}\;-\;}[\frac{E_{a}}{R}\cdot (\frac{1}{T}-\frac{1}{T_{ref}})]$$

where *E_a_* is the activation energy of parameter *k_s_*, *k_s,Tref_* is the deterioration rate constant at reference temperature *T_ref_*, and *R* is the universal gas constant. The shelf life was then determined by the overall sensory acceptance as a function of storage temperature as follows (Equation (4)):

(4) $$SL=\frac{S_{0}-S_{L}}{k_{{s,T}_{ref}}\cdot \exp\left(-\frac{E_{a}}{R}\left(\frac{1}{T}-\frac{1}{T_{ref}}\right)\right)}$$

where *SL* is the acceptance limit of sensory parameter *S* fixed to 5.

### 3.6. Statistical Analysis

The results were expressed as means ± standard deviation of three experimental replicates. For the estimation of the main interaction effects of the investigated factors, a factorial analysis of variance (factorial ANOVA) was used. As a post hoc analysis for the separation of means with significant differences (*p* < 0.05), Duncan’s multiple range test was used. For all statistical analyses, the Statistica 7 software (StatSoft, Tulsa, OK, USA) package was used. For all the mathematical regressions, the IBM SPSS Statistics Version 19 software package (IBM Corporation, Armonk, NY, USA) was used, and R^2^ and standard errors of model parameters were calculated.

## 4. Conclusions

This research investigated the formulation, development, and comprehensive quality evaluation of innovative, shelf-stable olive paste dip prototypes (OPD and OPD_Enr_), enriched with antioxidant compounds and exhibiting functional characteristics. The aim was to valorize agri-food by-products derived from tomato processing and olive oil production BACs, specifically total carotenoids (TC) from tomato pomace and phenolic compounds (TPC) from olive pomace, that were incorporated into the olive paste dip OPD_Enr_. Quantitative analysis revealed that the carotenoid content, phenolic content and antioxidant activity of OPD_Enr_ were higher compared to the corresponding values of OPD. Both OPD_Enr_ and OPD products were rated highly in sensory evaluation, particularly in terms of color and texture, achieving overall acceptability scores of 8.0/9.0 (OPD_Enr_) and 9.0/9.0 (OPD). However, the shelf life estimations, based on overall sensory acceptability, ranged from 65 to 110 days for OPD_Enr_ and from 74 to 281 days for OPD at temperatures from 20 to 40 °C. The research findings suggest that both OPD_Enr_ and OPD represent promising, health-promoting functional food products, offering an effective approach to the upcycling of tomato and olive oil industry by-products. Nevertheless, further research related to the in vitro bioaccessibility of the incorporated bioactive compounds in the matrix of olive paste dips needs to be conducted so as to validate the health-promoting profile of the proposed food prototype.

## Figures and Tables

**Table 1:** Physicochemical, nutritional and quality properties of the developed olive paste dip products OPD and OPD_Enr_.

Physicochemical/Quality Property	Product	
	OPD	OPD_Enr_
Fibers, total dietary (g/10 0 g)	10.0 ± 0.8	
Nitrogen content (g/100 g)	0.49 ± 0.04	
Proteins (g/100 g)	3.1 ± 0.3	
Lipids (g/100 g)	15.4 ± 0.5	
Saturated	2.73 ± 0.21	
Monounsaturated	10.6 ± 0.83	
Polyunsaturated	2.01 ± 0.18	
Carbohydrates (g/100 g)	13.1	
Sugars, total (g/100 g)	1.26 ± 0.12	
Sodium (mg Na/100 g)	410 ± 50	
Salt content (g/100 g)	1.03 ± 0.13	
Total energy (Kcal/100 g)	223	
pH	3.984 ± 0.007 ^a^	4.059 ± 0.008 ^a^
Ash content (g/100 g)	1.40 ± 0.1 ^a^	1.50 ± 0.11 ^a^
Moisture content (g/100 g)	54.2 ± 0.06 ^a^	55.2 ± 0.4 ^a^
Water activity (a_w_)	0.898 ± 0.090 ^a^	0.908 ± 0.091 ^a^
Titratable acidity (ΤA%)	3.980 ± 0.018 ^a^	4.845 ± 0.193 ^b^
Total phenolic content (TPC) (mg GAE/g)	1.62 ± 0.08 ^a^	3.05 ± 0.10 ^b^
Antioxidant capacity (AA) (mg Trolox/g)	0.801 ± 0.075 ^a^	0.976 ± 0.032 ^b^
Total carotenoids (TC) (mg/kg)	20.0 ± 2.00 ^a^	30.2 ± 1.06 ^b^
Color (CIE, *L* parameter)	16.6 ± 2.01 ^a^	19.8 ± 0.21 ^b^
Color (CIE, *a* parameter)	7.47 ± 0.63 ^a^	8.64 ± 0.11 ^b^
Color (CIE, *b* parameter)	13.9 ± 1.73 ^a^	18.0 ± 0.01 ^b^
Texture (firmness) (*n*)	0.839 ± 0.093 ^a^	0.427 ± 0.040 ^b^
Score of overall sensory quality	9.0 ± 0.0 ^a^	8.0 ± 0.2 ^b^

Different superscript small letters indicate significant differences between mean ± standard deviation as calculated by Duncan’s multiple range test for a significance level of *p* = 0.05.

**Table 2:** Evolution of TMVC during storage at 20, 30 and 40 °C. Products: OPD and OPD_Enr._

TMVC (logCFU/g)								
Storage Time (d)	20 °C			30 °C			40 °C	
	OPD	OPD_Enr_		OPD	OPD_Enr_		OPD	OPD_Enr_
0	2.76 ± 0.14 ^aA^	3.17 ± 0.27 ^aA^	0	2.76 ± 0.14 ^aA^	3.17 ± 0.27 ^aA^	0	2.76 ± 0.14 ^aA^	3.17 ± 0.27 ^aA^
24	3.41 ± 0.59 ^aA^	3.30 ± 0.15 ^aA^	24	3.16 ± 0.04 ^aA^	3.00 ± 0.45 ^aA^	24	2.93 ± 0.22 ^aA^	<2.00
64	3.56 ± 0.23 ^aA^	3.30 ± 0.23 ^aA^	64	3.24 ± 0.34 ^aA^	3.05 ± 0.13 ^aA^	64	2.89 ± 0.80 ^aA^	<2.00
119	3.12 ± 0.28 ^aA^	3.43 ± 0.59 ^aA^	106	3.18 ± 025 ^aA^	3.12 ± 0.34 ^aA^	106	2.30 ± 0.11 ^aA^	<2.00

Mean ± standard error. Different superscript small letters in the same column indicate significant differences between means, indicating the effect of storage time at each temperature on TMVC as calculated by Duncan’s multiple range test for a significance level of *p* = 0.05. Different superscript capital letters in the same row indicate significant differences between means, indicating the effect of olive paste product on TMVC as calculated by Duncan’s multiple range test for a significance level of *p* = 0.05.

**Table 3:** Quality degradation rates (based on total sensory quality, k_s,_ d^−1^) for the products, OPD and OPD_Enr_, at 20, 30, 40 °C.

Product	OPD		OPD_Enr_	
T (°C)	*k_s_* (d^−1^)	R^2^	*k_s_* (d^−1^)	R^2^
20	0.0166 ± 0.0047 ^aA^	0.896	0.0264 ± 0.0041 ^bA^	0.976
30	0.0363 ± 0.0033 ^aB^	0.984	0.0439 ± 0.0086 ^aB^	0.895
40	0.0461 ± 0.0072 ^aB^	0.871	0.0535 ± 0.0115 ^aB^	0.806
*E_a_* (kJ/mol) k_ref_(d^−1^) (T_ref_ = 25°C)	39.2 ± 11.2 ^a^ 0.0236 ± 0.0028 ^a^	0.924	27.2 ± 6.33 ^a^ 0.0333 ± 0.0035 ^b^	0.948

Mean ± standard error. Different superscript small letters in the same column indicate significant differences between means, indicating the effect of storage temperature on ks as calculated by Duncan’s multiple range test for a significance level of *p* = 0.05. Different superscript capital letters in the same row indicate significant differences between means, indicating the effect of olive paste product on ks as calculated by Duncan’s multiple range test for a significance level of *p* = 0.05.

**Table 4:** Olive paste dip product formulation (serving size was 100 g of net product weight). Products: OPD and OPD_Enr._

Ingredient	Product (g/100 g)	
	OPD	OPD_Enr_
Dry, debittered olive pomace	16.7	16.7
Pepper	15.0	15.0
Olive rings	11.1	11.1
Glycerol	10.0	10.0
Pomace oil	10.0	7.5
BAC_S_	_	10.0
Maltodextrin	7.5	_
Vinegar	7.0	7.0
Tomato	3.0	3.0
Caper	3.0	3.0

The encapsulation system contained the following ingredients per 100 g: wall material: 20 g maltodextrin, 1 g CMC (wall material, nanoemulsion: 10 g pomace oil, 8 g Tween 80, 2.3 g olive extract and capsules: 60 g maltodextrin, 30 g oil (1.85 g tomato extract), 7 g olive extract and 3 g CMC.

## Data Availability

The data presented in this study are available on request from the corresponding authors.

## Supplementary Materials

The following supporting information can be downloaded at: https://www.mdpi.com/article/10.3390/molecules30173459/s1. Table S1: Tested sensory characteristics for the olive paste dip product.

## References

[1] Allison B.J., Simmons C.W. (2017). Valorization of tomato pomace by sequential lycopene extraction and anaerobic digestion. Biomass Bioenergy.

[2] Viuda-Martos M., Sanchez-Zapata E., Sayas-Barberá E., Sendra E., Pérez-Álvarez J.A., Fernández-López J. (2014). Tomato and tomato byproducts. human health benefits of lycopene and its application to meat products: A Review. Crit. Rev. Food Sci. Nutr..

[3] Liadakis G., Katsouli M., Chanioti S., Giannou V., Tzia C. (2022). Chapter One: Identification, quantification, and characterization of tomato processing by-products. Tomato Processing by-Products. Sustainable Applications.

[4] European Environment Agency (2009). Diverting Waste from Landfill: Effectiveness of Waste Management Policies in the European Union.

[5] Abbattista R., Ventura G., Calvano C.D., Cataldi T.R.I., Losito I. (2021). Bioactive compounds in waste by-products from olive oil production: Applications and structural characterization by mass spectrometry techniques. Foods.

[6] European Commission Agriculture and Rural Development. Olive Oil in the EU..

[7] Skaltsounis A.L., Argyropoulou A., Aligiannis N., Xynos N., Boskou D. (2015). 11—Recovery of high added value compounds from olive tree products and olive processing byproducts. Olive and Olive Oil Bioactive Constituents.

[8] Chanioti S., Tzia C. (2017). Optimization of ultrasound-assisted extraction of oil from olive pomace using response surface technology: Oil recovery, unsaponifiable matter, total phenol content and antioxidant activity. LWT.

[9] Tsevdou M., Ntzimani A., Katsouli M., Dimopoulos G., Tsimogiannis D., Taoukis P. (2024). Comparative study of microwave, pulsed electric fields, and high pressure processing on the extraction of antioxidants from olive pomace. Molecules.

[10] Trombino S., Cassano R., Procopio D., Di Giola M.L., Barone E. (2021). Valorization of tomato waste as a source of carotenoids. Molecules.

[11] Strati I.F., Oreopoulou V. (2014). Recovery of carotenoids from tomato processing by-products—A review. Food Res. Int..

[12] Difonzo G., Troilo M., Squeo G., Pasqualone A., Caponio F. (2021). Functional compounds from olive pomace to obtain high-added value foods—A review. J. Sci. Food Agric..

[13] Katsouli M., Thanou I.V., Raftopoulou E., Ntzimani A., Taoukis P., Giannakourou M.C. (2024). Bioaccessibility and stability Studies on encapsulated phenolics and carotenoids from olive and tomato pomace: Development of a functional fruit beverage. Appl. Sci..

[14] Araújo M., Pimentel F.B., Alves R.C., Oliveira M.B.P. (2015). Phenolic compounds from olive mill wastes: Health effects, analytical approach and application as food antioxidants. Trends Food Sci. Technol..

[15] Zbakh H., El Abbassi A. (2012). Potential use of olive mill wastewater in the preparation of functional beverages: A review. J. Funct. Foods.

[16] Andreou V., Chanioti S., Stergiou P., Katsaros G. (2021). Valorization of the olive oil production residue: Healthy ingredient for developing high value-added spread. Sustainability.

[17] Roselló-Soto E., Koubaa M., Moubarik A., Lopes R.P., Saraiva J.A., Boussetta N., Grimi N., Barba F.J. (2015). Emerging oppor-tunities for the effective valorization of wastes and by-products generated during olive oil production process: Non-conventional methods for the recovery of high-added value compounds. Trends Food Sci. Technol..

[18] Meng Q., Long P., Zhou J., Ho C.T., Zou X., Chen B., Zhang L. (2019). Improved Absorption of β-carotene by encapsulation in an oil-in-water nanoemulsion containing tea polyphenols in the aqueous phase. FoodRes. Int..

[19] Ezhilarasi P.N., Karthik P., Chhanwal N., Chinnaswamy A. (2013). Nanoencapsulation techniques for food bioactive components: A review. Food Bioproc.Technol..

[20] Pérez-Andrés J.M., Charoux C.M., Cullen P.J., Tiwari B.K. (2018). Chemical modifications of lipids and proteins by nonthermal food processing technologies. J. Agric. Food Chem..

[21] Benshitrit R.C., Levi C.S., Tal S.L., Shimoni E., Lesmes U. (2012). Development of oral food-grade delivery systems: Current knowledge and future challenges. Food Funct..

[22] Chen S., Han Y., Jian L., Liao W., Zhang Y., Gao Y. (2020). Fabrication, characterization, physicochemical stability of zein-chitosan nanocomplex for co-encapsulating curcumin and resveratrol. Carbohydr. Polym..

[23] Han Y., Pei Y., Wang J., Xiao Z., Miao Y., Wang Z., Zhang F., Hou W., Yi Y., Chen S. (2024). Research progress on the nano-delivery systems of food-derived bioactive components. Food Biosci..

[24] Shin G.H., Kim J.T., Park H.J. (2015). Recent developments in nanoformulations of lipophilic functional foods. Trends Food Sci. Technol..

[25] Liu Q., Huang H., Chen H., Lin J., Wang Q. (2019). Food-grade nanoemulsions: Preparation, stability and application in encapsulation of bioactive compounds. Molecules.

[26] Aboalnaja K.O., Yaghmoor S., Kumosani T.A., Mc Clements D.J. (2016). Utilization of nanoemulsions to enhance bioactivity of pharmaceuticals, supplements, and nutraceuticals: Nanoemulsion delivery systems and nanoemulsion excipient systems. Expert. Opin. Drug Deliv..

[27] Fang Z., Bhandari B. (2010). Encapsulation of polyphenols-a review. Trends Food Sci. Technol..

[28] da Silva Soares B., de Carvalho C.W.P., Garcia-Rojas E.E. (2021). Microencapsulation of sacha inchi oil by complex coacervates using ovalbumin-tannic acid and pectin as wall materials. Food Bioproc. Technol..

[29] Favaro-Trindade C.S., Patel B., Silva M.P., Comuniana T.A., Federici E., Jones O.G., Campanella O.H. (2020). Microencapsulation as a tool to producing an extruded functional food. LWT.

[30] Göktepe S., Ocak B., Özdestan Ö. (2021). Physico-chemical, sensory, and antioxidant characteristics of olive paste enriched with microencapsulated thyme essential oil. Food Bioproc. Technol..

[31] Lanza B., Di Serio M.G., Giansante L., Di Loreto G., Russi F., Di Giacinto L. (2013). Effects of pasteurisation and storage on quality characteristics of table olives preserved in olive oil. Int. J. Food Sci. Technol..

[32] Cosmai L., Campanella D., Summo C., Paradiso V.M., Pasqualone A., De Angelis M., Caponio F. (2017). Combined effects of a natural Allium spp. extract and modified atmo spheres packaging on shelf life extension of olive-based paste. Int. J. Food Sci. Technol..

[33] Cosmai L., Campanella D., De Angelis M., Summo C., Paradiso V.M., Pasqualone A., Caponio F. (2018). Use of starter cultures for table olives fermentation as possibility to improve the quality of thermally stabilized olive-based paste. LWT.

[34] Nanis I., Hatzikamari M., Katharopoulos E., Boukouvala E., Ekateriniadou E., Litopoulou-Tzanetaki E., Gerasopoulos D. (2020). Microbiological and physicochemical changes during fermentation of solid residue of olive mill wastewaters: Exploitation towards the production of an olive paste–type product. LWT.

[35] Schwartz M., Quitral V., Daccarett C., Callejas J. (2009). Development of spreadable olive paste from the Sevillana variety. Grasas Y Aceites.

[36] Choi M.H., Kim G.H., Lee H.S. (2002). Effects of ascorbic acid retention on juice color and pigment stability in blood orange (*Citrus sinensis*) juice during refrigerated storage. Food Res. Int..

[37] Stoll L., Costa T.M.H., Jablonski A., Flôres S.H., Rios A.O. (2016). Microencapsulation of anthocyanins with different wall materials and its application in active biodegradable films. Food Bioproc. Technol..

[38] Jafari S.M., Ghanbari V., Dehnad D., Ganje M. (2021). Improving the storage stability of tomato paste by the addition of encapsu lated olive leaf phenolics and experimental growth modeling of *A. flavus*. Int. J. Food Microbiol..

[39] Talhaoui N., Taamalli A., Gómez-Caravaca A.M., Fernández Gutiérrez A., Segura-Carretero A. (2015). Phenolic compounds in olive leaves: Analytical determination, biotic and abiotic influence, and health benefits. Food Res. Int..

[40] Karagozlu M., Ocak B., Özdestan-Ocak Ö. (2021). Effect of tannic acid concentration on the physicochemical, thermal, and anti oxidant properties of gelatin/gum Arabic–walled microcapsules containing *Origanum onites* L. essential oil. Food Bioproc. Technol..

[41] Mahungu S.M., Diaz-Mercado S., Li J., Schwenk M., Singletary K., Faller J. (1999). Stability of isoflavones during extrusion processing of corn/soy mixture. J. Agric. Food Chem..

[42] Erskine E., Özkan G., Lu B., Capanoglu E. (2023). Effects of fermentation process on the antioxidant capacity of fruit byproducts. ACS Omega.

[43] Martins S.I.F.S., Jongen W.M.F., van Boekel M.A.J.S. (2000). A review of Maillard reaction in food and implications to kinetic modelling. Trends Food Sci. Technol..

[44] Bolchini S., Larcher R., Morozova K., Scampicchio M., Nardin T. (2024). Screening of Antioxidant Maillard Reaction Products Using HPLC-HRMS and Study of Reaction Conditions for Their Production as Food Preservatives. Molecules.

[45] Cam M., Icyer N.C., Erdogan F. (2014). Pomegranate peel phenolics: Microencapsulation, storage stability and potential ingredient for functional food development. LWT.

[46] Lanza B., Amoruso F. (2016). Sensory analysis of natural table olives: Relationship between appearance of defect and gustatory-kinaesthetic sensation changes. LWT.

[47] Gotoh N., Wada S. (2006). The importance of peroxide value in assessing food quality and food safety. J. Amer Oil Chem. Soc..

[48] Pleasance E.A., Kerr W.L., Pegg R.B., Swanson R.B., Cheely A.N., Huang G., Parrish D.R., Kerrihard A.L. (2018). Effects of storage conditions on consumer and chemical assessments of raw ‘nonpareil’almonds over a two-year period. J. Food Sci..

[49] Karaaslan M., Şengün F., Cansu Ü., Başyiğit B., Sağlam H., Karaaslan A. (2021). Gum arabic/maltodextrin microencapsulation confers peroxidation stability and antimicrobial ability to pepper seed oil. Food Chem..

[50] Lanza B., Di Serio M.G., Iannucci E., Russi F., Marfisi P. (2009). Nutritional, textural and sensorial characterisation of Italian table olives (*Olea europaea* L. cv. ‘Intosso d’Abruzzo’). Int. J. Food Sci. Technol..

[51] European Food Safety Authority (2008). Scientific Opinion of the Panel on Food Additives, Flavourings, Processing Aids and Materials in Contact with Food on a request from the Commission on the safety in use of lycopene as a food colour. EFSA J..

[52] Ramirez E., García-García P., de Castro A., Romero C., Brenes M. (2013). Debittering of black dry-salted olives. Eur. J. Lipid Sci. Technol..

[53] Tamer C.E., Incedayı B., Yıldız B., Çopur Ö.U. (2013). The Use of vacuum impregnation for debittering green olives. Food Bioprocess Technol..

[54] Latimer G.W., AOAC (1995). Loss on Drying (Moisture) in Nuts and Nut Products. Official Methods of Analysis of AOAC International.

[55] Horwitz W., AOAC (2000). Fat (Crude) in Nuts and Nut Products: Gravimetric methods. Official Methods of Analysis AOAC International.

[56] Latimer G.W., AOAC (2023). Fat (Crude) or Ether Extract in Animal Feed. Official Methods of Analysis AOAC International.

[57] Latimer G.W., AOAC (2023). Fat (Total, Saturated, and Unsaturated) in Foods: Hydrolytic Extraction Gas Chromatographic Method. Official Methods of Analysis of AOAC International.

[58] (2009). Food and Feed Products-General Guidelines for the Determination of Nitrogen by the Kjeldahl Method, 2nd ed. https://www.iso.org/obp/ui/#iso:std:iso:1871:ed-2:v1:en.

[59] Latimer G.W., AOAC (2023). 991.43 Total, Soluble, and insoluble dietary fiber in foods: Enzymatic-gravimetric method, MES-TRIS Buffer. Official Methods of Analysis of AOAC International.

[60] United States Environmental Protection Agency (1982). Method 613: 2,3,7,8-Tetrachlorodibenzo-p-Dioxin by Capillary Column GC/MS. Compendium of Methods for the Determination of Toxic Organic Compounds in Ambient Air.

[61] United States Environmental Protection Agency (1994). Method 200.8: Determination of Trace Elements in Waters and Wastes by Inductively Coupled Plasma–Mass Spectrometry, Revision 5.4.

[62] Katsouli M., Giannou V., Tzia C. (2020). Enhancement of physicochemical and encapsulation stability of O1/W/O2 multiple na noemulsions loaded with coenzyme Q10 or conjugated linoleic acid by incorporating polyphenolic extract. Food Funct..

[63] Bancuta O.R., Chilian A., Bancuta I., Ion R.M., Setnescu R., Setnescu T., Gheboianu A. (2016). Improvement of spectrophotometric method for determination of phenolic compounds by statistical investigations. Rom. Journ. Phys..

[64] Lichtenthaler H.K., Buschmann C. (2001). Chlorophylls and Carotenoids: Measurement and Characterization by UV-VIS Spectroscopy. Curr. Protoc. Food Anal. Chem..

[65] (2003). Peroxide Value-Acetic Acid-Chloroform Method.

[66] (2022). Sensory Analysis—General Guidance for the Staff of a Sensory Evaluation Laboratory—Part 1: Staff Responsibilities.

[67] (2022). Sensory Analysis—General Guidance for the Staff of a Sensory Evaluation Laboratory—Part 2: Recruitment and Training of Panel Leaders.

[68] (2017). Sensory Analysis—General Guidance for the Design of Test Rooms.

