# Supplementary material for: Design, Production and Quality Assessment of Antioxidant-Enriched Olive Paste Dips Using Agro-Food By-Products
Source: Molecules. 2025 Aug 22;30(17):3459. doi: 10.3390/molecules30173459 (PMC12430177; doi:10.3390/molecules30173459)
Supplement: Supplementary file 1 [file molecules-30-03459-s001.zip › molecules-3779245-supplementary.pdf]

Table S1 Tested sensory characteristics for the olive paste dip product.

**Characteristic**	**Adopted scale**
Characteristics pertaining to appearance	
Intensity of oil	Intensity scale 1-9
Intensity of homogeneity	Intensity scale 1-9
Intensity of color (light to dark)	Intensity scale 1-9
Characteristics pertaining to texture	
Intensity of firmness	Intensity scale 1-9
Intensity of juiciness	Intensity scale 1-9
Intensity of oily texture	Intensity scale 1-9
Characteristics pertaining to taste	
Intensity of sweet taste	Intensity scale 1-9
Intensity of salty taste	Intensity scale 1-9
Intensity of bitter taste	Intensity scale 1-9
Intensity of acidic taste	Intensity scale 1-9
Characteristics pertaining to aroma/flavor	
Intensity of earthy aroma/flavor	Intensity scale 1-9
Intensity of aroma/flavor	Intensity scale 1-9
Intensity of olive aroma/flavor	Intensity scale 1-9
Intensity of tomato pepper aroma/flavor	Intensity scale 1-9
Other aroma/flavor	Intensity scale 1-9
Intensity of rancidity	Intensity scale 1-9
Product appearance liking	Hedonic scale 1-9
Product odor liking	Hedonic scale 1-9
Product taste liking	Hedonic scale 1-9
Product aftertaste liking	Hedonic scale 1-9
Overall sensory quality/liking	Hedonic scale 1-9
